# Author Correction: Assessment of human leukocyte antigen-based neoantigen presentation to determine pan-cancer response to immunotherapy

**DOI:** 10.1038/s41467-025-57244-4

**Published:** 2025-02-24

**Authors:** Jiefei Han, Yiting Dong, Xiuli Zhu, Alexandre Reuben, Jianjun Zhang, Jiachen Xu, Hua Bai, Jianchun Duan, Rui Wan, Jie Zhao, Jing Bai, Xuefeng Xia, Xin Yi, Chao Cheng, Jie Wang, Zhijie Wang

**Affiliations:** 1 State Key Laboratory of Molecular Oncology, CAMS Key Laboratory of Translational Research on Lung Cancer, Department of Medical Oncology, National Cancer Center/National Clinical Research Center for Cancer/Cancer Hospital, Chinese Academy of Medical Sciences and Peking Union Medical College, Beijing, 100021 China; 2 Department of Neuro-oncology, Neurosurgery Center, Beijing Tiantan Hospital, Capital Medical University, Beijing, China; 3 Geneplus-Beijing Institute, Beijing, China; 4 University of Chinese Academy of Sciences, Beijing, 100049 China; 5 CAS Key Laboratory of Genomic and Precision Medicine, Beijing Institute of Genomics, Chinese Academy of Sciences and China National Center for Bioinformation, Beijing, 100101 China; 6 Department of Thoracic/ Head and Neck Medical Oncology, The University of Texas M. D. Anderson Cancer Center, Houston, Texas USA; 7 Department of Medicine, Dan L Duncan Comprehensive Cancer Center, Institute for Clinical and Translational Research, Baylor College of Medicine, Houston, Texas USA

Correction to: *Nature Communications* 10.1038/s41467-024-45361-5, published online 8 February 2024

In the version of the article initially published, the middle panel of Fig. 3f was a duplicate of the left panel and has now been corrected in the HTML and PDF versions of the article.

