# Supplementary material for: Assessment of human leukocyte antigen-based neoantigen presentation to determine pan-cancer response to immunotherapy
Source: Nat Commun. 2024 Feb 8;15:1199. doi: 10.1038/s41467-024-45361-5 (PMC10853168; doi:10.1038/s41467-024-45361-5)
Supplement: Supplementary file 1 — Supplementary Information [file 41467_2024_45361_MOESM1_ESM.pdf]

SUPPLEMENTARY INFORMATION

Supplementary Figure 1. HLA and TNB between homozygotes and heterozygotes.

Supplementary Figure 2. Distribution of HLA-I divergence, TNB, and log10(TNB+1).

Supplementary Figure 3. Distribution of HLA-I Antigen Presentation Score (HAPS) values in each cohort.

Supplementary Figure 4. Median value of the HLA-I Antigen Presentation Score (HAPS) in all immune checkpoint inhibitor (ICI)-treated patients.

Supplementary Figure 5. In vitro validation of functional T cells stimulated by predicted neoantigens.

Supplementary Figure 6. Efficacy of the HLA-I Antigen Presentation Score (HAPS) in predicting overall survival (OS) with immune checkpoint inhibitors (ICIs) in each cohort.

Supplementary Figure 7. Comparison of predictive value of HLA-I Antigen Presentation Score (HAPS) based on NetMHCpan4.0 and NetMHCpan4.1

Supplementary Figure 8. Single Cox regression analysis in ICI-treated patients.

Supplementary Figure 9. Correlation of TNB & HAPS with tumor stage.

Supplementary Figure 10. Efficacy of the HLA-I Antigen Presentation Score (HAPS) compared with TNB and HLA divergence in predicting overall survival (OS) with immune checkpoint inhibitors (ICIs) in NSCLC and SKCM

Supplementary Figure 11. Relationship between HLA-I Antigen Presentation Score (HAPS) and treatment response.

Supplementary Figure 12. Relationship between HLA-I Antigen Presentation Score (HAPS) and tumor mutation burden/programmed death ligand 1 (TMB/PD-L1).

Supplementary Figure 13. TMB/TNB based on the gene panel and whole-exome sequencing (WES).

Supplementary Figure 14. Patients stratified according to loss of heterozygosity (LOH) and HLA-I Antigen Presentation Score (HAPS) in each cohort.

Supplementary Figure 15. Immune cell subsets analyzed via single-sample gene set enrichment analysis (ssGSEA) based on RNA-seq data

Supplementary Figure 16. Relationship between HLA-I Antigen Presentation Score (HAPS) and dynamic evolution of the TCR repertoire.

Supplementary Figure 17. Varied predictive values of nine factors for individual patient.

Supplementary Figure 18. The architecture of the neural network model.

Supplementary Figure 19. Clinical responses of individuals predicted by a neural network model.

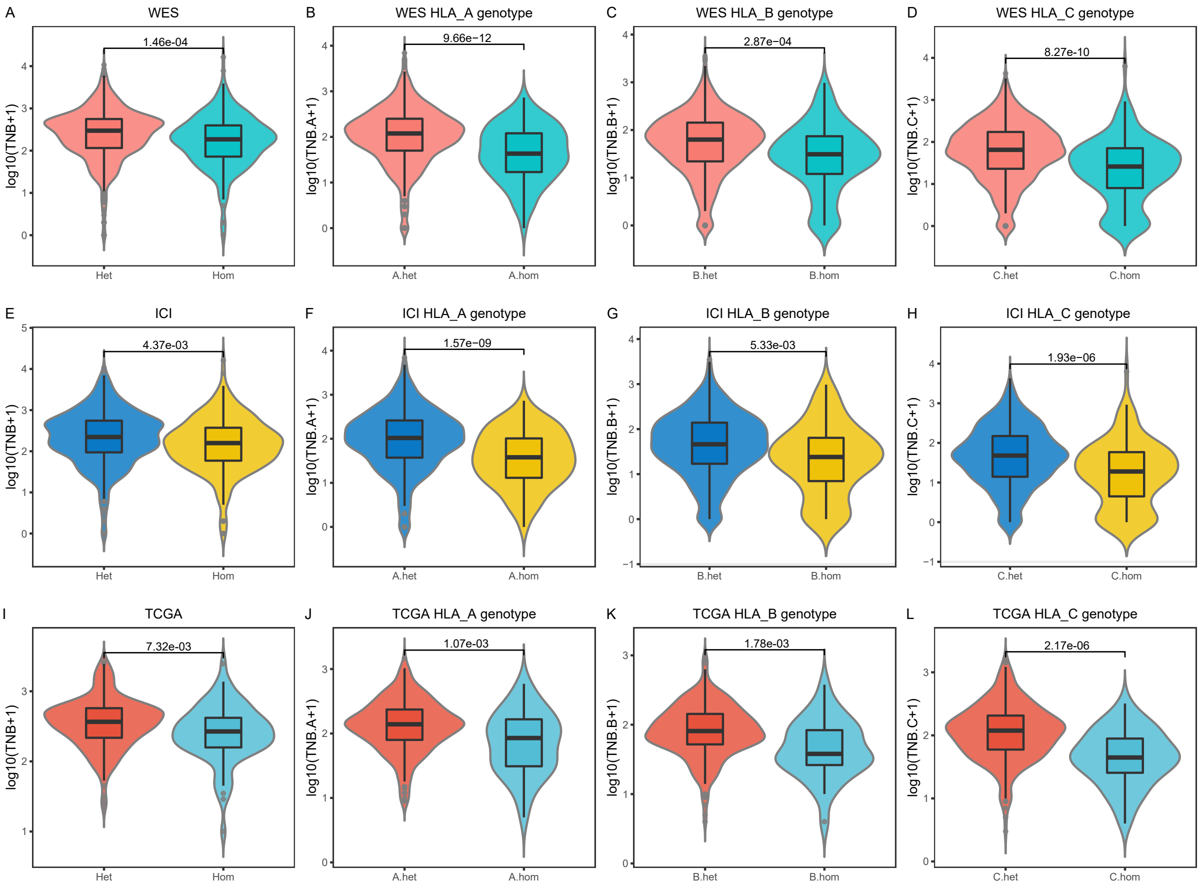

Het, heterozygotes. Hom, homozygotes. WES, whole-exome sequencing. ICI, immune checkpoint inhibitors.

Supplementary Figure 1. TNB between homozygotes and heterozygotes

Left: Distribution of TNB for each *HLA* genotype (left) and log10(TNB+1) (right) between homozygotes and heterozygotes across all patients (N=1,125) (10 immune checkpoint inhibitor-treated cohorts and The Cancer Genome Atlas database, respectively). Source data are provided as a Source Data file.

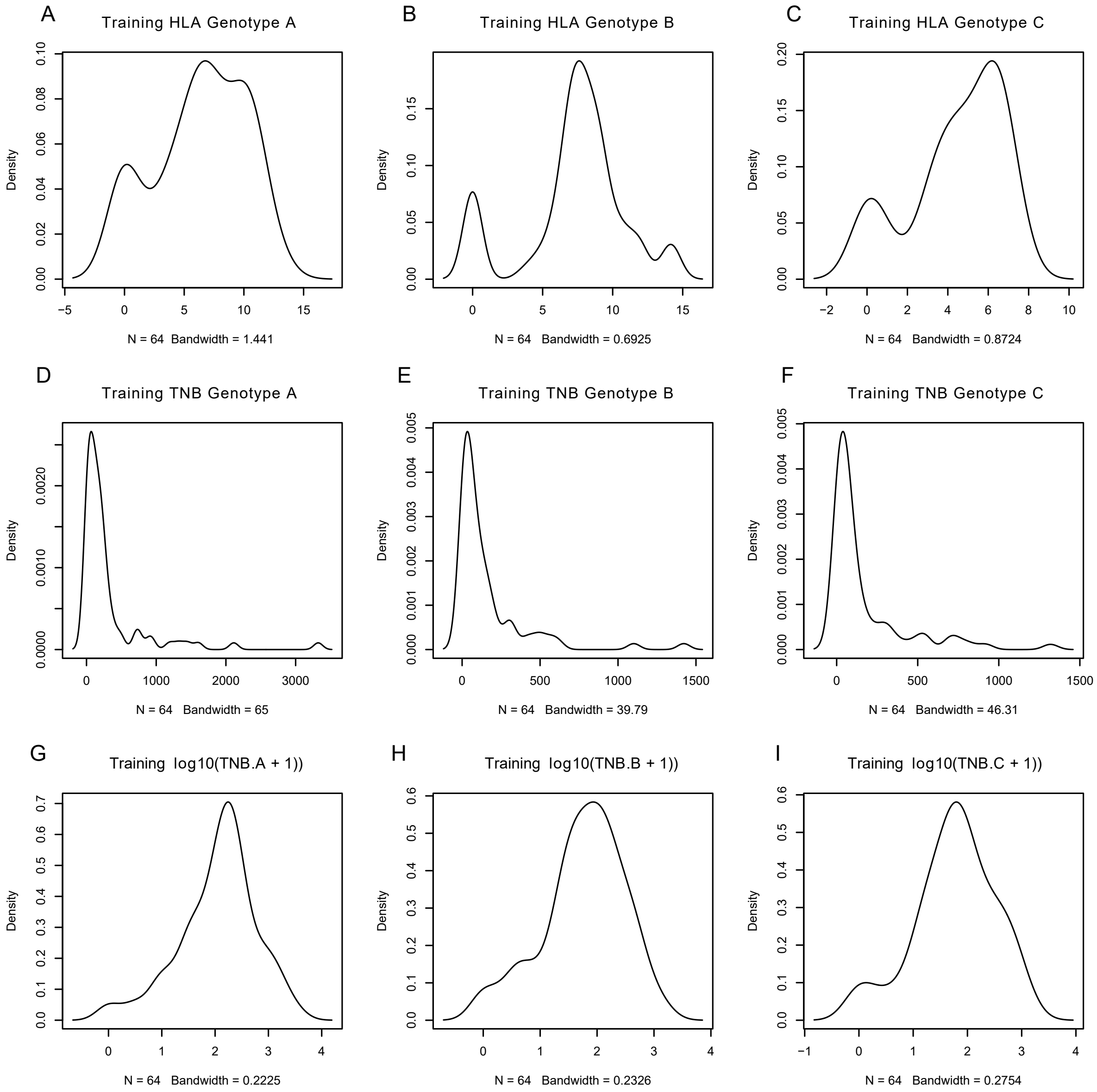

Supplementary Figure 2. Distribution of *HLA-I* divergence, TNB, and log10(TNB+1).

Distribution of HLA-I divergence, TNB, and log10(TNB+1) among *HLA* genotypes in the training set (N=64). Source data are provided as a Source Data file.

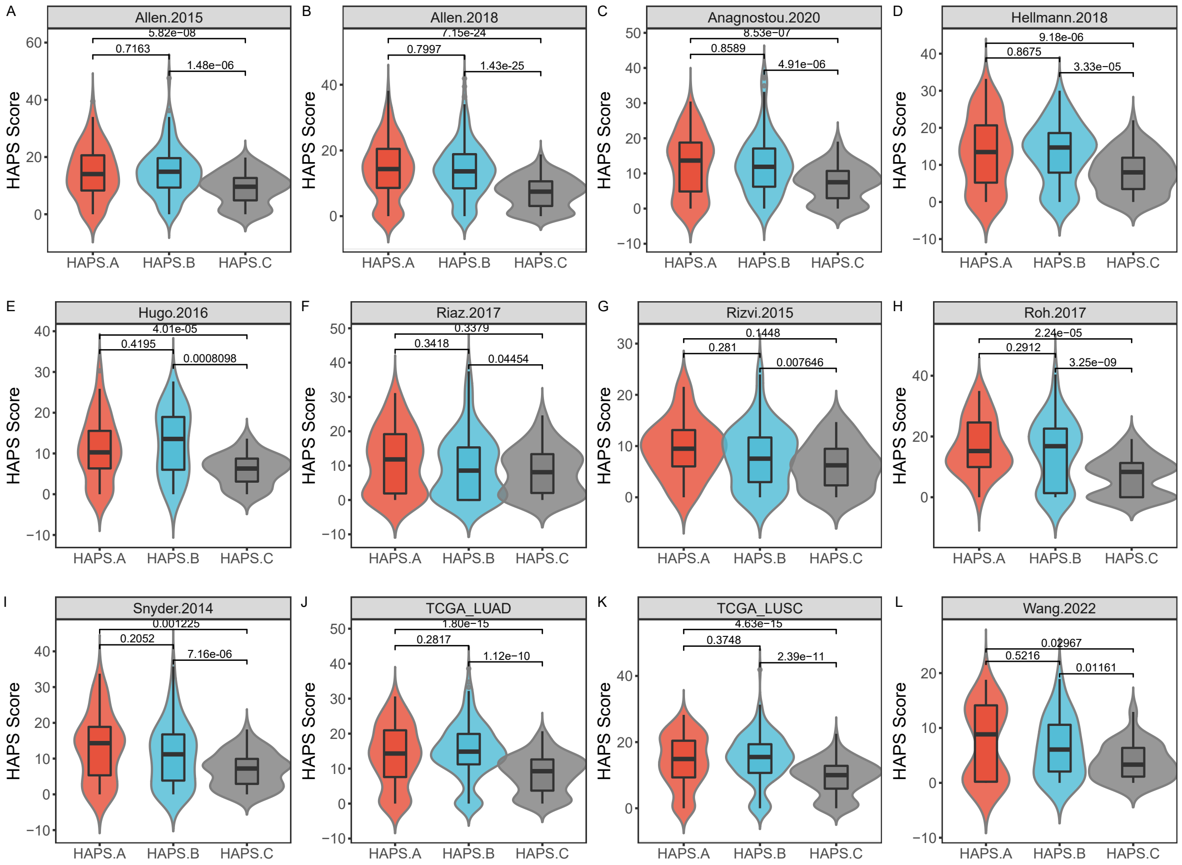

Supplementary Figure 3. Distribution of *HLA-I* Antigen Presentation Score (HAPS) values in each cohort

Distribution of HAPS among *HLA* genotypes in 12 cohorts with whole-exome sequencing data (N=1125). Source data are provided as a Source Data file.

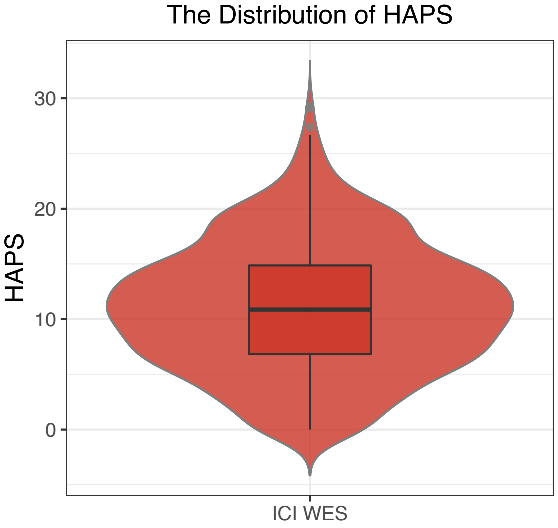

Supplementary Figure 4. Median value of the *HLA-I* Antigen Presentation Score (HAPS) in all immune checkpoint inhibitor (ICI)-treated patients

Distribution and median value of the HAPS in ICI-treated patients (N=792). Source data are provided as a Source Data file.

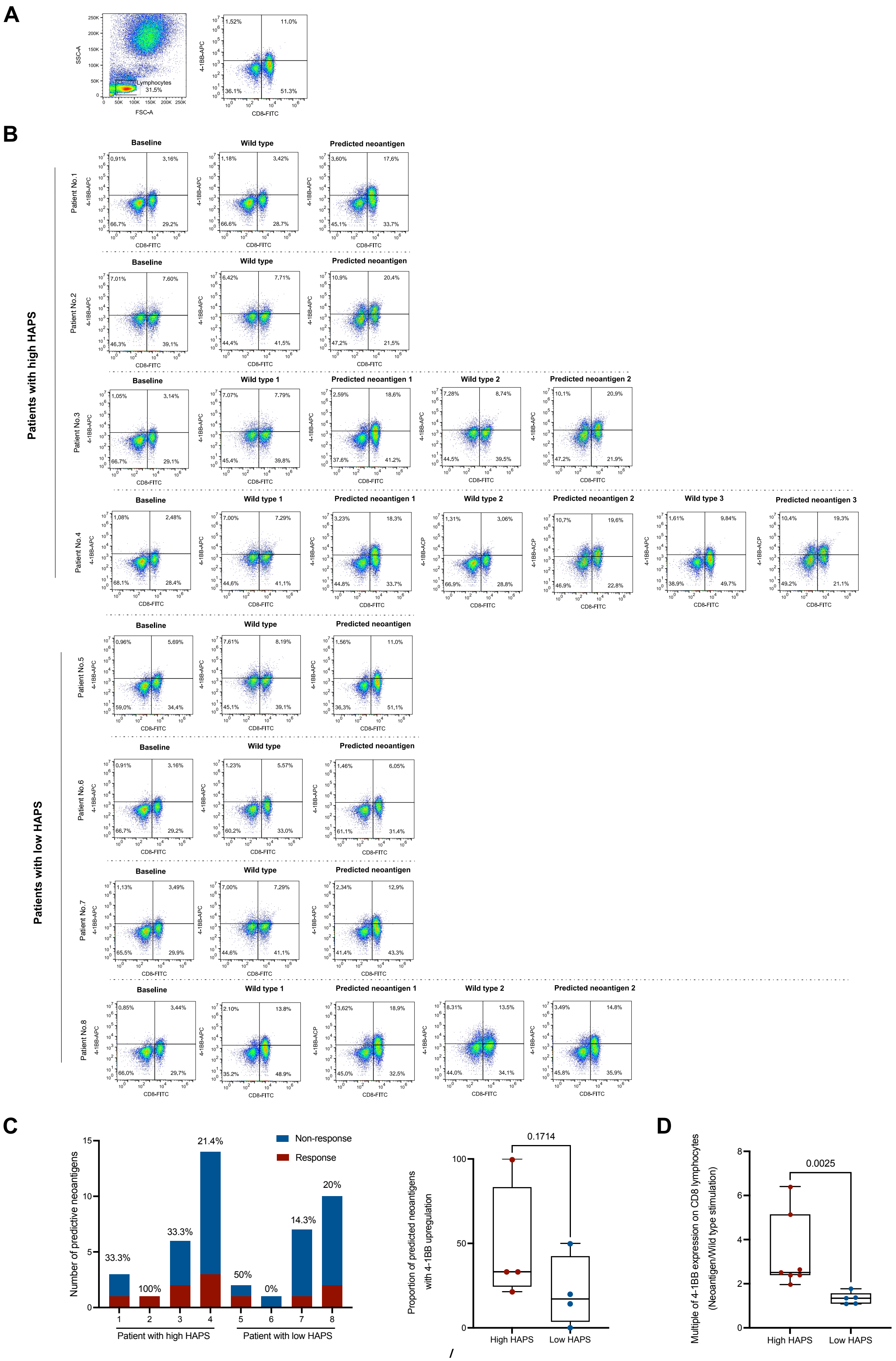
A
SSC-A
FSC-A
Lymphocytes
31.5%
4-1BB-APC
CD8-FITC
1.52%
11.0%
36.1%
51.3%
B
Patients with high HAPS
Patients with low HAPS
Patient No.1
Patient No.2
Patient No.3
Patient No.4
Patient No.5
Patient No.6
Patient No.7
Patient No.8
Baseline
Wild type
Predicted neoantigen
Wild type 1
Predicted neoantigen 1
Wild type 2
Predicted neoantigen 2
Wild type 3
Predicted neoantigen 3
C
Number of predictive neoantigens
Non-response
Response
33.3%
100%
33.3%
21.4%
50%
0%
14.3%
20%
Patient with high HAPS
Patient with low HAPS
Proportion of predicted neoantigens with 4-1BB upregulation
0.1714
High HAPS
Low HAPS
D
Multiple of 4-1BB expression on CD8 lymphocytes (Neoantigen/Wild type stimulation)
0.0025
High HAPS
Low HAPS

Supplementary Figure 5. In vitro validation of functional T cells stimulated by predicted neoantigens

A. Gating strategies for 4-1BB on CD8+ lymphocytes. B. Flow cytometry analysis for the expression of 4-1BB on CD8+ lymphocytes after co-culture with predicted neoantigens or wild-type counterparts in patients with high (upper) and low HAPS (lower). C. Proportion of predicted neoantigens leading to up-regulation of 4-1BB on CD8+ T cells in different groups. D. Higher activation of 4-1BB on CD8+ lymphocytes stimulated by predicted neoantigens in the high HAPS group than in the low HAPS group.

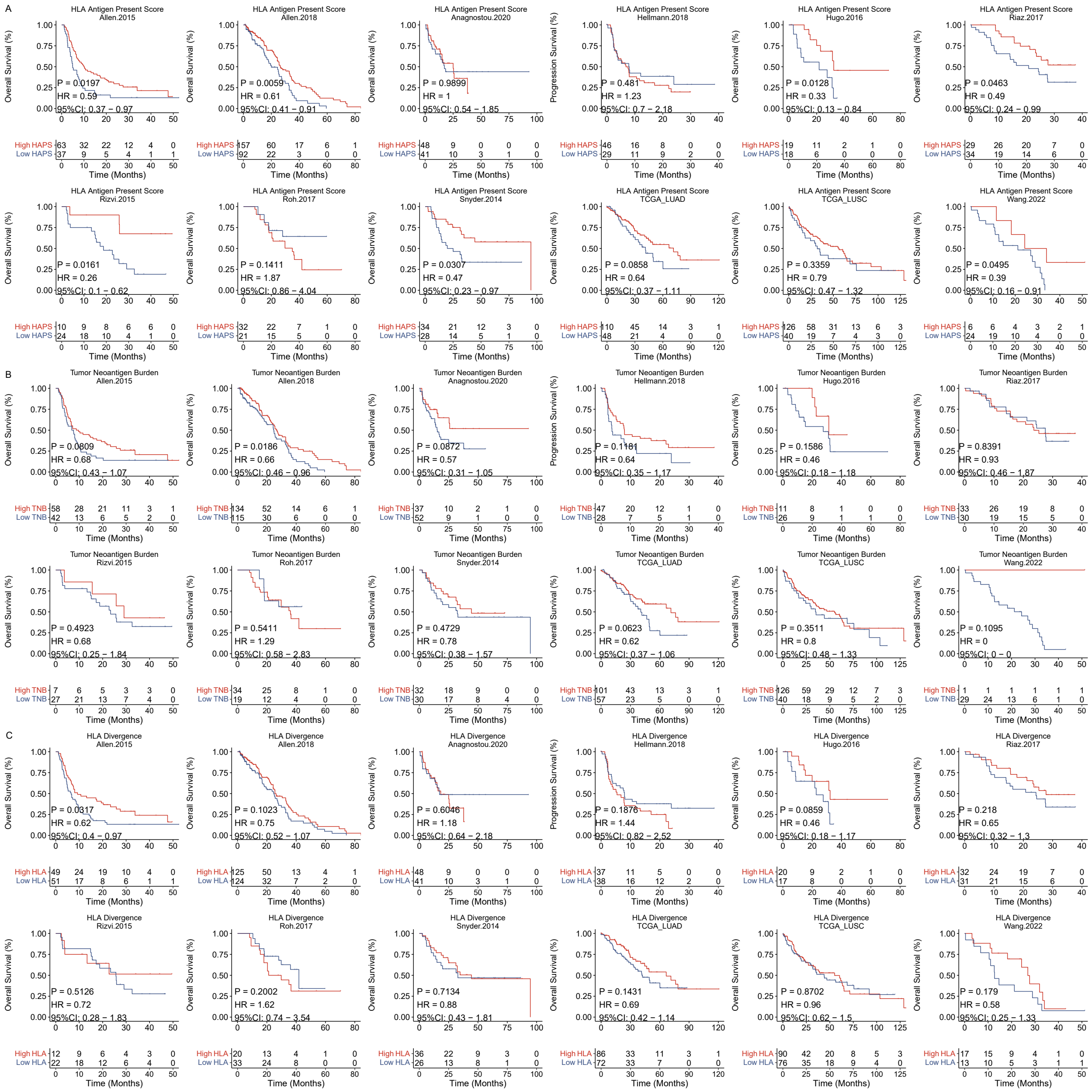

Supplementary Figure 6. Efficacy of the *HLA-I* Antigen Presentation Score (HAPS) compared with TNB and HLA divergence in predicting overall survival (OS) with immune checkpoint inhibitors (ICIs) in each cohort

Association of high HAPS (A), HLA divergence (B), and TNB (C) with OS after receiving ICIs in 12 cohorts with whole-exome sequencing data. Source data are provided as a Source Data file.

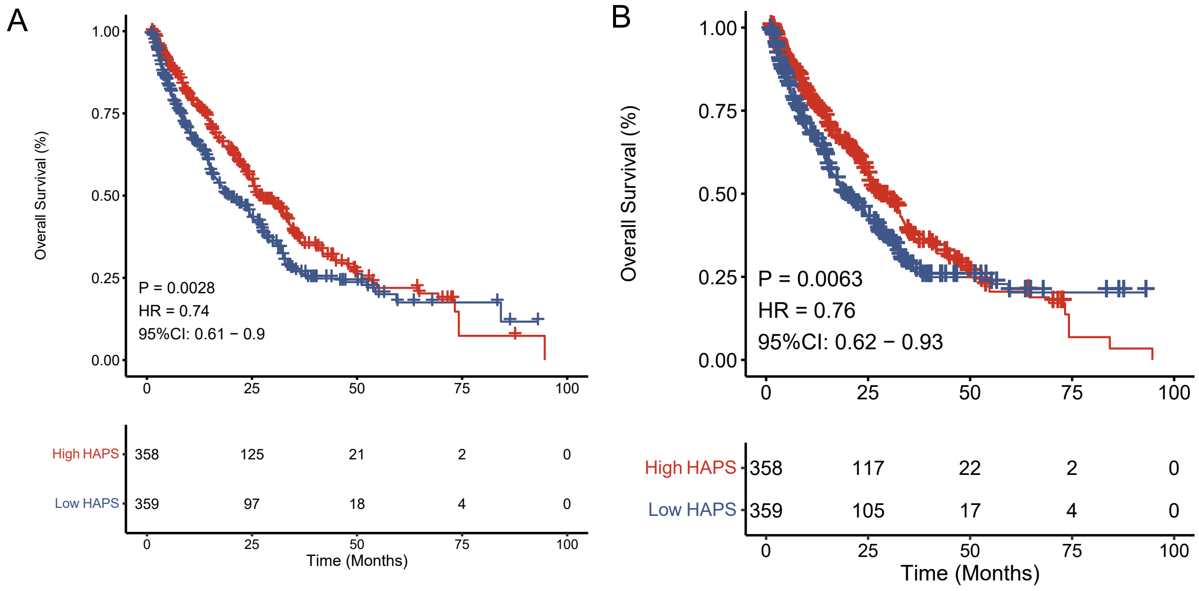

Supplementary Figure 7. Comparison of predictive value of HLA-I Antigen Presentation Score (HAPS) based on NetMHCpan4.0 and NetMHCpan4.1

Overall survival of immune checkpoint inhibitor (ICI) stratified by HAPS based on IC50 (NetMHCpan4.0) (A) and rank 1% (NetMHCpan4.1) (B). Source data are provided as a Source Data file.

Single Cox Analysis	Pvalue	HR	95%CI
TMB >14 VS <=14	2.14E−01	0.71	[0.42,1.22]
PDL1 >=1% VS <1%	5.88E−01	0.89	[0.58,1.37]
Stage IV VS III	1.95E−01	1.14	[0.93,1.39]
Age >65 VS <=65	3.25E−01	0.85	[0.62,1.17]
Gender Male VS Female	2.20E−01	0.88	[0.72,1.08]
HLA Divergence	4.16E−01	0.98	[0.94,1.03]
TNB	7.90E−03	1.00	[1.00,1.00]
HAPS High VS Low	2.27E−04	0.69	[0.57,0.84]

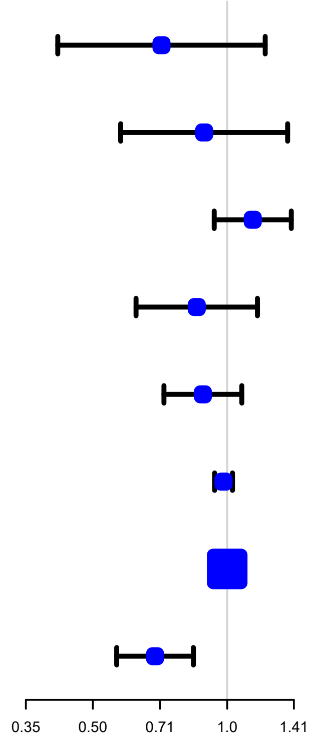

Supplementary Figure 8. Single Cox regression analysis in ICI-treated patients

The single Cox regression analysis of tumor mutation burden, programmed death ligand 1, age, gender, HLA divergence, TNB, and *HLA-I* Antigen Presentation Score in ICI-treated patients (N=717). Source data are provided as a Source Data file.

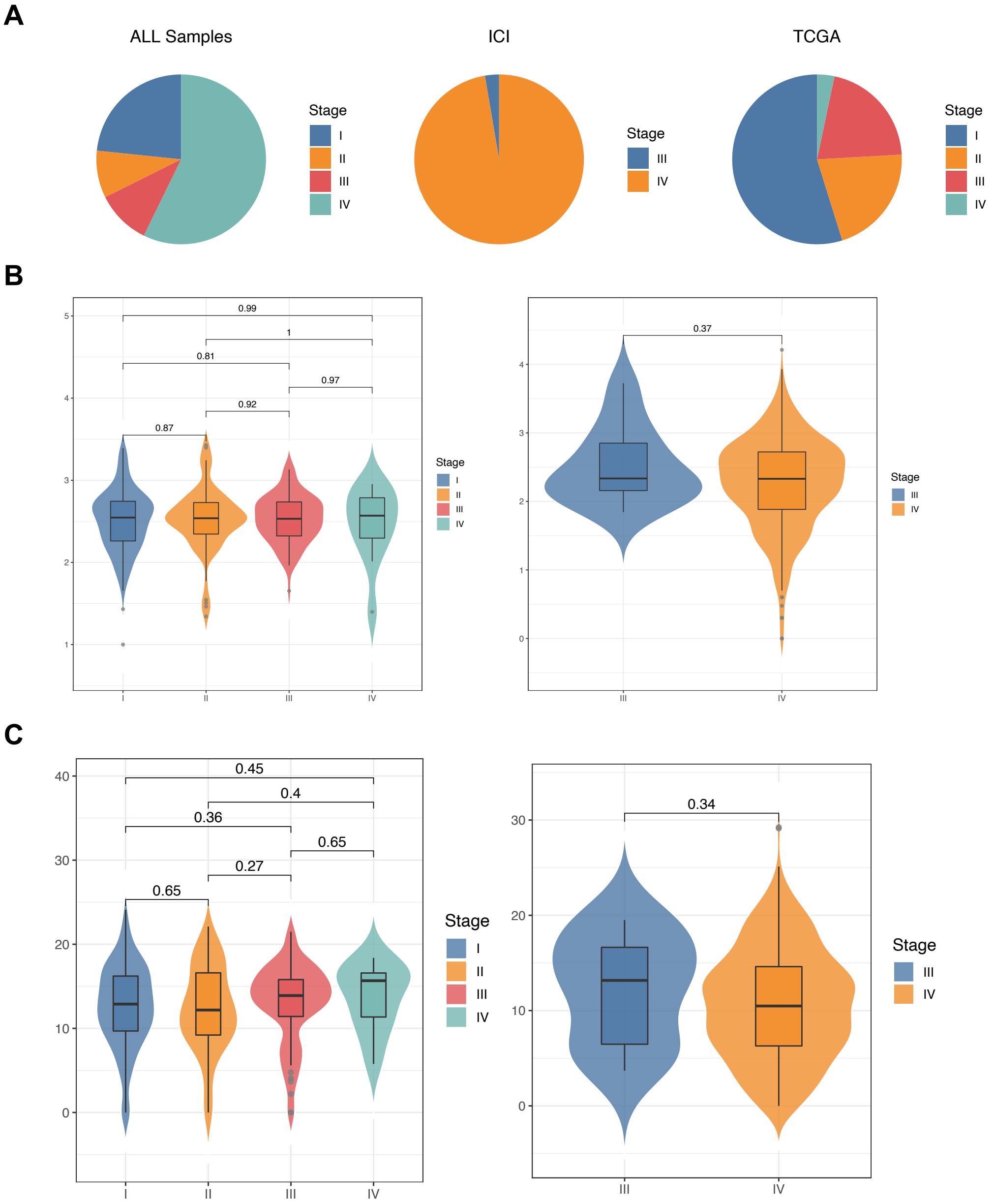

Supplementary Figure 9. Correlation of TNB & HAPS with tumor stage.

A. Distribution of tumor stage in ICI and TCGA cohort (N=779). C. Distribution of TNB according to tumor stage; B. Distribution of TNB according to tumor stage in TCGA (left) or ICI (cohort); C. Distribution of HAPS according to tumor stage in TCGA (left) or ICI (cohort). Source data are provided as a Source Data file.

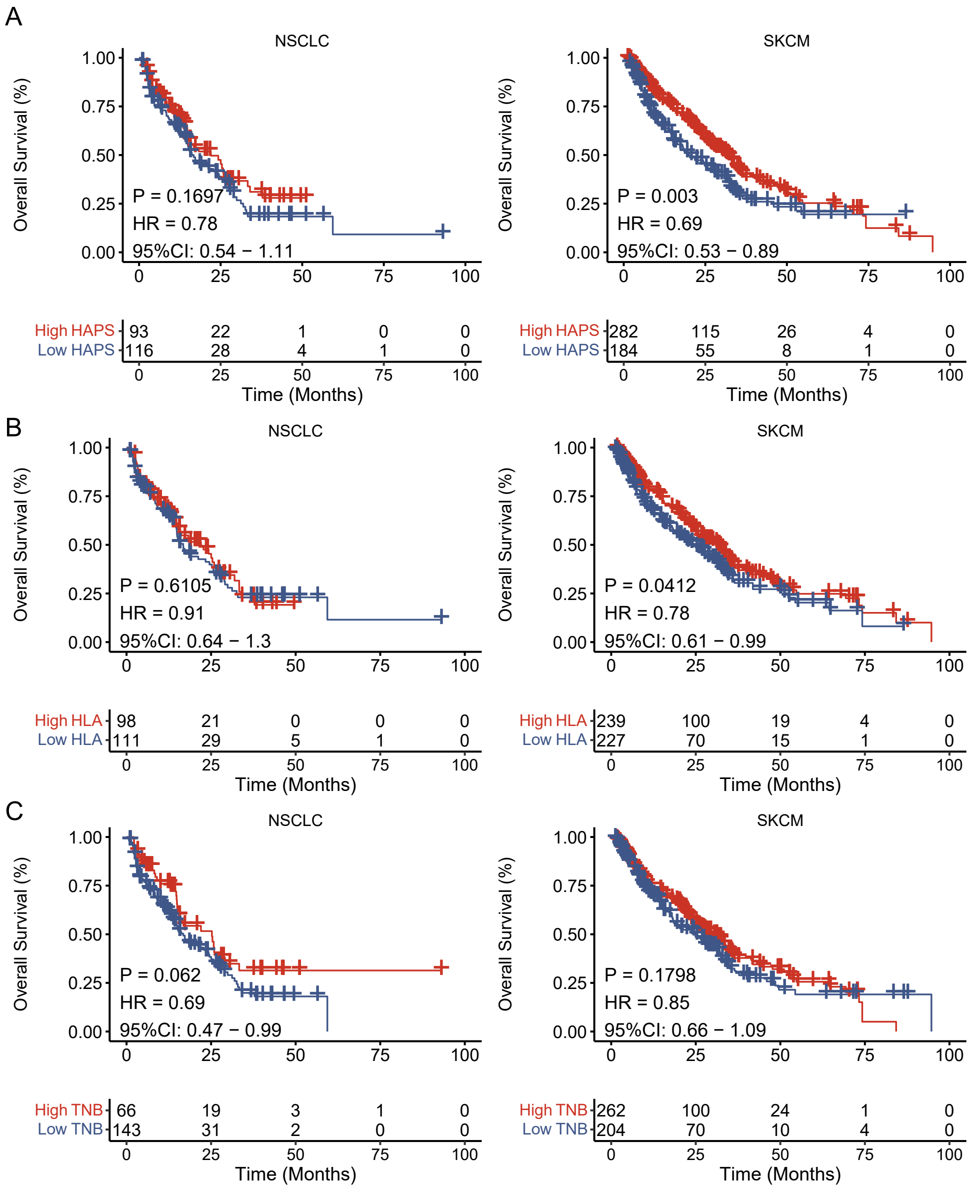

Supplementary Figure 10. Efficacy of the *HLA-I* Antigen Presentation Score (HAPS) compared with TNB and HLA divergence in predicting overall survival (OS) with immune checkpoint inhibitors (ICIs) in NSCLC and SKCM

Association of high HAPS (A), HLA divergence (B), and TNB (C) with OS after receiving ICIs in NSCLC and SKCM. Source data are provided as a Source Data file.

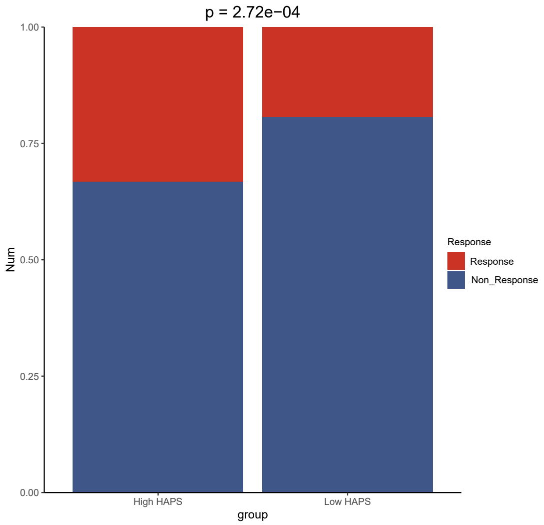

Supplementary Figure 11. Relationship between *HLA-I* Antigen Presentation Score (HAPS) and treatment response

Statistically significant differences in response rate between high and low HAPS subgroups (N=562). Source data are provided as a Source Data file.

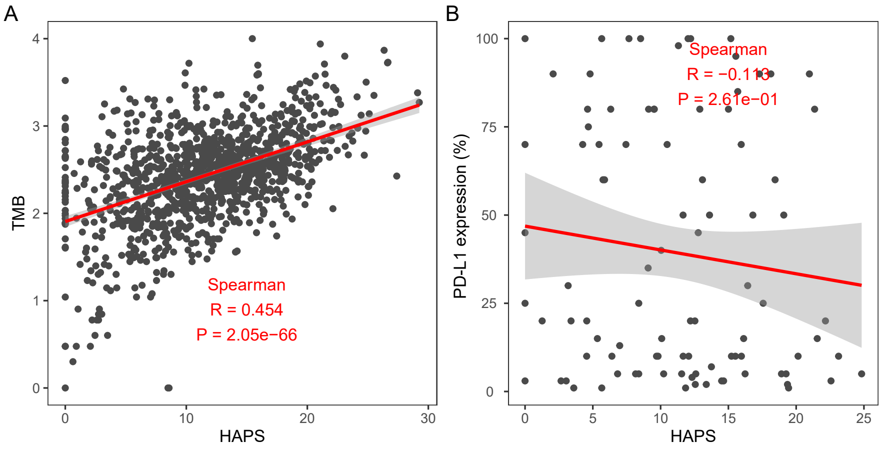

Supplementary Figure 12. Relationship between *HLA-I* Antigen Presentation Score (HAPS) and tumor mutation burden/programmed death ligand 1 (TMB/PD-L1)

Relationship between HAPS and TMB (left)/PD-L1(right) (N=1125). Source data are provided as a Source Data file.

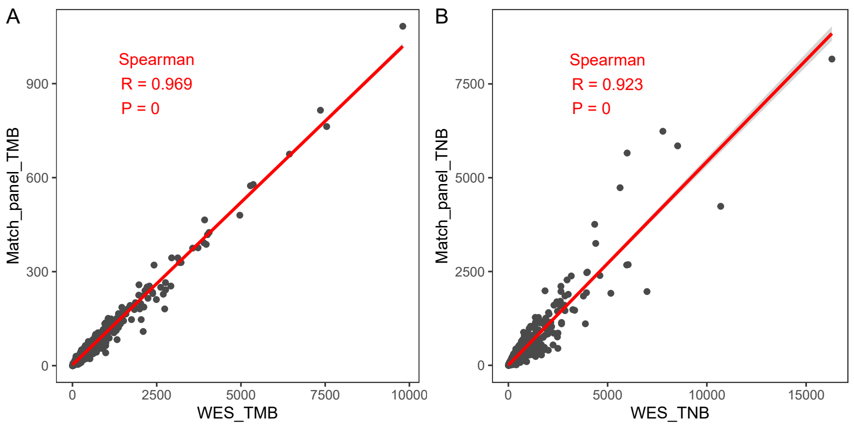

Supplementary Figure 13. TMB/TNB based on the gene panel and whole-exome sequencing (WES).

Left: the relationship between panel- and WES-based TMB. Right: the relationship between panel- and WES- based TNB (N=1125). Source data are provided as a Source Data file.

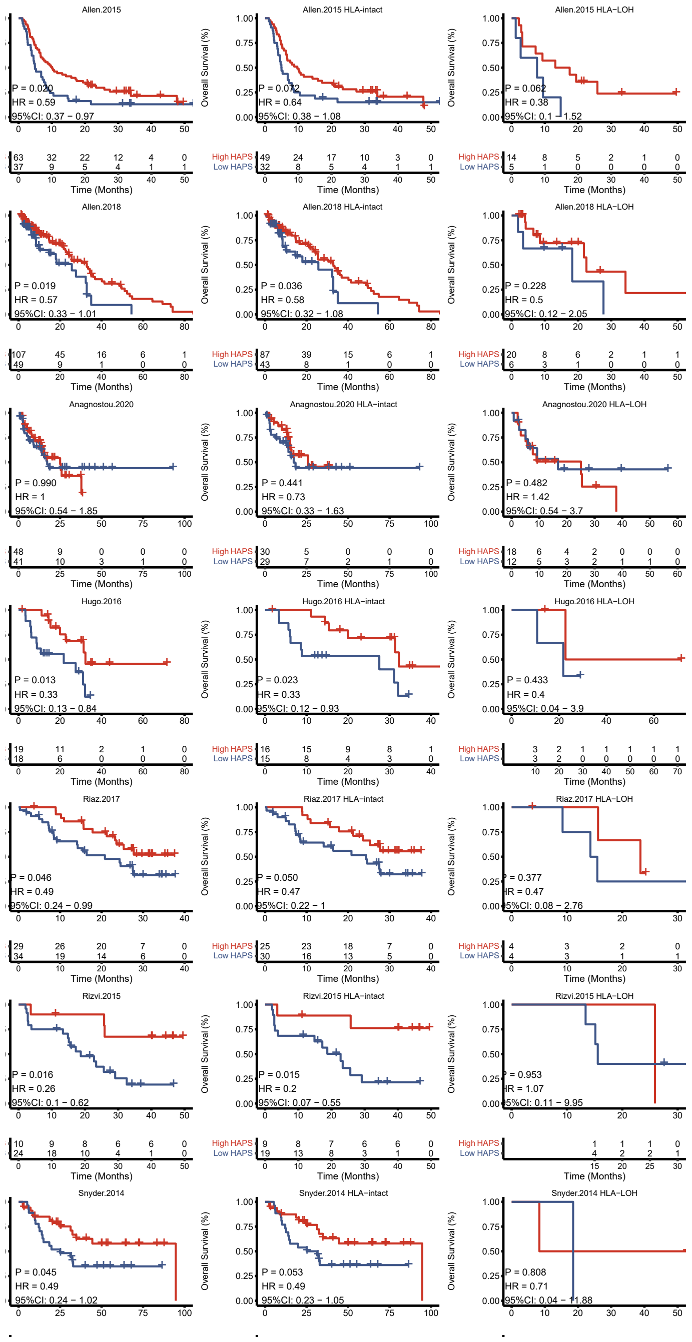

Supplementary Figure 14. Patients stratified according to loss of heterozygosity (LOH) and *HLA-I* Antigen Presentation Score (HAPS) in each cohort

Differences in overall survival between high and low HAPS in all patients (left), patients with intact HLA (middle), and patients with HLA-LOH (right) in eight cohorts. Source data are provided as a Source Data file.

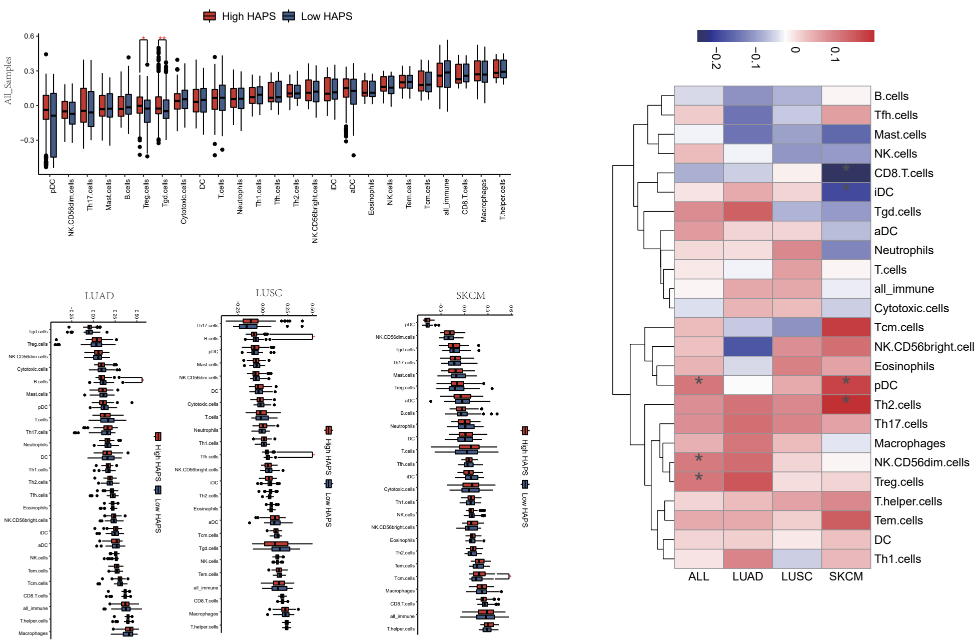

Supplementary Figure 15. Immune cell subsets analyzed via single-sample gene set enrichment analysis (ssGSEA) based on RNA-seq data

Differences in specific immune cell subsets between high and low HAPS among all lung adenocarcinoma (LUAD), lung squamous cell carcinoma (LUSC), and skin cutaneous melanoma (SKCM) patients with RNA-seq data, respectively. Heatmap of specific immune cell subsets among all LUAD, LUSC, and SKCM patients with RNA-seq data (N=455). Source data are provided as a Source Data file.

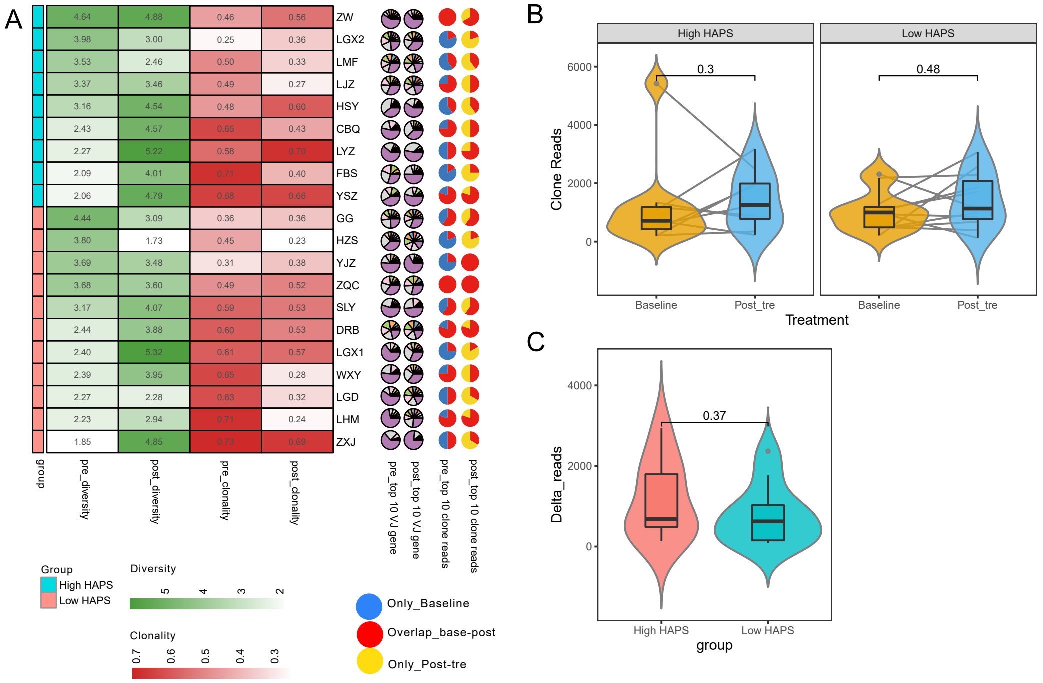

Supplementary Figure 16. Relationship between *HLA-I* Antigen Presentation Score (HAPS) and dynamic evolution of the TCR repertoire.

This analysis is based on patients from the Wang-Blood cohort. **(A)** Individual changes in diversity and clonality pre- and post-treatment. **(B-C)** Number of T cell receptor (TCR) clone reads pre- and post-treatment in the high and low HAPS groups. Source data are provided as a Source Data file.

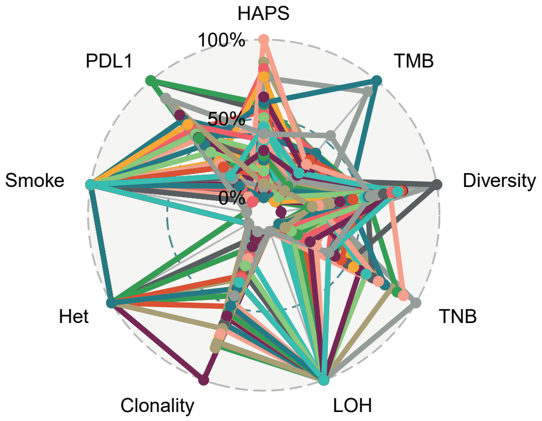

Supplementary Figure 17. Varied predictive values of nine factors for individual patient.

Each colored trajectory corresponds to a distinct patient, and the dots along this trajectory denote the relative influence of each factor on the therapeutic response of that particular patient. Source data are provided as a Source Data file.

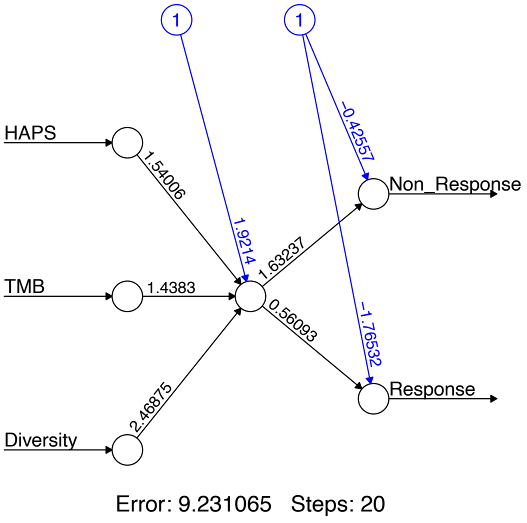

Supplementary Figure 18. The architecture of the neural network model.

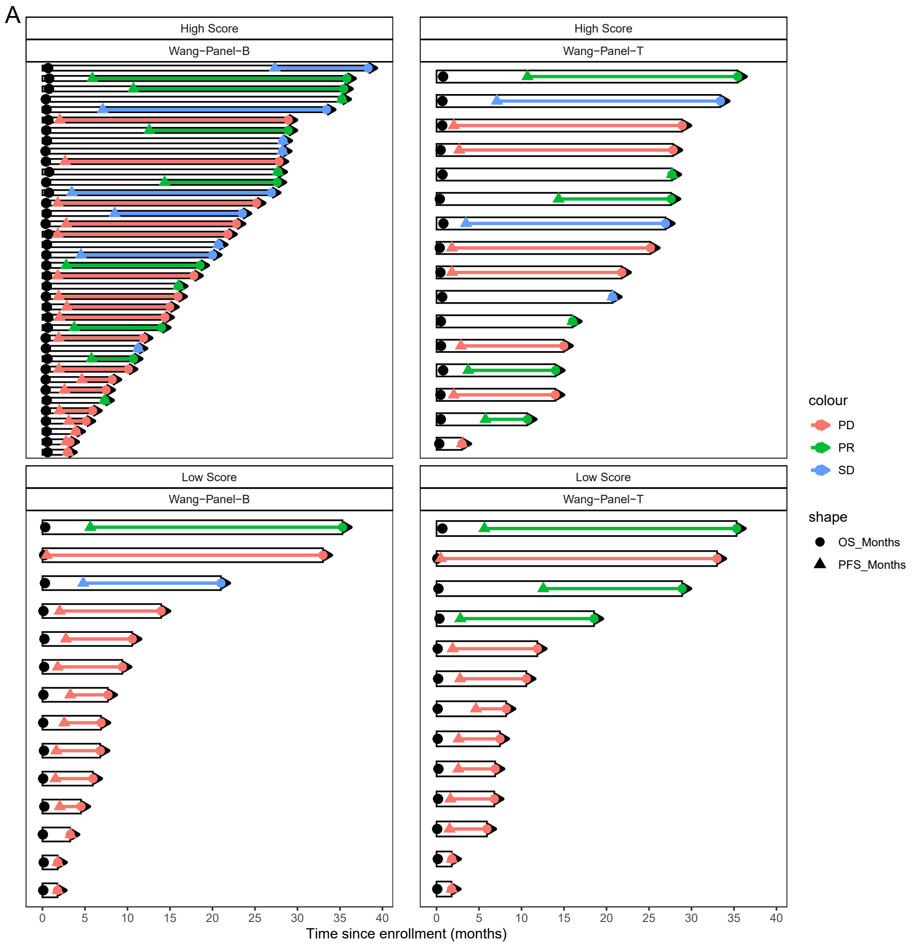

Supplementary Figure 19. Clinical responses of individuals predicted by a neural network model

Swimming map exhibiting overall survival/progression-free survival months and progressive disease/partial response/stable disease responses pre- and post-treatment in the Wang-Tissue and Wang-Blood cohorts. Source data are provided as a Source Data file.
