# Supplementary material for: Assessment of human leukocyte antigen-based neoantigen presentation to determine pan-cancer response to immunotherapy
Source: Nat Commun. 2024 Feb 8;15:1199. doi: 10.1038/s41467-024-45361-5 (PMC10853168; doi:10.1038/s41467-024-45361-5)
Supplement: Supplementary file 3 — Description of Additional Supplementary Files [file 41467_2024_45361_MOESM3_ESM.pdf]

Description of Additional Supplementary Files

File Name: Supplementary Data 1
Description: Patient characteristics.

File Name: Supplementary Data 2
Description: Amino acid sequences of predicted neoantigens.

File Name: Supplementary Data 3
Description: Predictive value of HAPS in enrolled patients.

File Name: Supplementary Data 4
Description: Gene list of the NGS-based 1,021 panel

File Name: Supplementary Data 5
Description: Association of HAPS with response to immunotherapy stratified by HLA-LOH

File Name: Supplementary Data 6
Description: TME signature and function.

File Name: Supplementary Data 7
Description: Distribution of immune cells analyzed via ssGSEA.

File Name: Supplementary Data 8
Description: TCR and panel-based HAPS analysis of Wang-panel-T/B cohort.

File Name: Supplementary Data 9
Description: Data used in the neural network model.

File Name: Supplementary Data 10
Description: List of primer sequences used for PCR
