# Supplementary material for: Assessment of human leukocyte antigen-based neoantigen presentation to determine pan-cancer response to immunotherapy
Source: Nat Commun. 2024 Feb 8;15:1199. doi: 10.1038/s41467-024-45361-5 (PMC10853168; doi:10.1038/s41467-024-45361-5)
Supplement: Supplementary file 14 — Reporting Summary [file 41467_2024_45361_MOESM14_ESM.pdf]

nature portfolio

Corresponding author(s): Chao Cheng, Jie Wang and Zhijie Wang

Last updated by author(s): Dec 20, 2023

Reporting Summary

Nature Portfolio wishes to improve the reproducibility of the work that we publish. This form provides structure for consistency and transparency in reporting. For further information on Nature Portfolio policies, see our Editorial Policies and the Editorial Policy Checklist.

Statistics

For all statistical analyses, confirm that the following items are present in the figure legend, table legend, main text, or Methods section.

n/a	Confirmed	
☐	☒	The exact sample size (*n*) for each experimental group/condition, given as a discrete number and unit of measurement
☐	☒	A statement on whether measurements were taken from distinct samples or whether the same sample was measured repeatedly
☐	☒	The statistical test(s) used AND whether they are one- or two-sided<br>*Only common tests should be described solely by name; describe more complex techniques in the Methods section.*
☐	☒	A description of all covariates tested
☐	☒	A description of any assumptions or corrections, such as tests of normality and adjustment for multiple comparisons
☐	☒	A full description of the statistical parameters including central tendency (e.g. means) or other basic estimates (e.g. regression coefficient) AND variation (e.g. standard deviation) or associated estimates of uncertainty (e.g. confidence intervals)
☐	☒	For null hypothesis testing, the test statistic (e.g. *F*, *t*, *r*) with confidence intervals, effect sizes, degrees of freedom and *P* value noted<br>*Give P values as exact values whenever suitable.*
☒	☐	For Bayesian analysis, information on the choice of priors and Markov chain Monte Carlo settings
☒	☐	For hierarchical and complex designs, identification of the appropriate level for tests and full reporting of outcomes
☐	☒	Estimates of effect sizes (e.g. Cohen's *d*, Pearson's *r*), indicating how they were calculated

Our web collection on statistics for biologists contains articles on many of the points above.

Software and code

Policy information about availability of computer code

Data collection	https://github.com/zxl2014swjx/HAPS.git
Data analysis	https://github.com/zxl2014swjx/HAPS.git

For manuscripts utilizing custom algorithms or software that are central to the research but not yet described in published literature, software must be made available to editors and reviewers. We strongly encourage code deposition in a community repository (e.g. GitHub). See the Nature Portfolio guidelines for submitting code & software for further information.

Data

Policy information about availability of data

All manuscripts must include a data availability statement. This statement should provide the following information, where applicable:

- Accession codes, unique identifiers, or web links for publicly available datasets
- A description of any restrictions on data availability
- For clinical datasets or third party data, please ensure that the statement adheres to our policy

All data needed to evaluate the conclusions of the current study are present in the manuscript and/or the Supplementary Materials. The source code and data associated with this paper are available at https://github.com/zxl2014swjx/HAPS.git. Whole-exome sequencing, T-cell repertoire sequencing and panel sequencing data have been deposited in Genome Sequence Archive under accession code PRJCA018167.

Research involving human participants, their data, or biological material

Policy information about studies with human participants or human data. See also policy information about sex, gender (identity/presentation), and sexual orientation and race, ethnicity and racism.

Reporting on sex and gender	This study did not include gender as a factor for analysis, as there is currently no research evidence to suggest that gender differences have an impact on the presentation ability of HLA antigens.
Reporting on race, ethnicity, or other socially relevant groupings	The patients included in this article are based on publicly available datasets that have been published, as well as patients enrolled at our center, and do not involve race, ethnicity, or other socially relevant groupings.
Population characteristics	The participant cohort in our study was characterized by a consistency in their past and current diagnoses and treatment categories.
Recruitment	The present study consists of patients from public datasets and patients from our center. The inclusion of patients from public datasets depended on which studies provided publicly available data that could be analyzed. The inclusion of patients from our center comprised all patients who received immunotherapy between March 14, 2017 and May 2, 2018. There is no self-selection bias or other biases that may impact the results
Ethics oversight	This study was approved by the ethics committees of the National Cancer Center/Cancer Hospital, Chinese Academy of [Medical Sciences, and the Peking Union Medical College (NCC2016JZ-03 and NCC2018-092). All enrolled patients provided written informed consent.

Note that full information on the approval of the study protocol must also be provided in the manuscript.

Field-specific reporting

Please select the one below that is the best fit for your research. If you are not sure, read the appropriate sections before making your selection.

☒ Life sciences ☐ Behavioural & social sciences ☐ Ecological, evolutionary & environmental sciences

For a reference copy of the document with all sections, see nature.com/documents/nr-reporting-summary-flat.pdf

Life sciences study design

All studies must disclose on these points even when the disclosure is negative.

Sample size	No sample-size calculation was performed. The present study included 792 patients from public datasets and 93 patients from our center. The inclusion of patients from public datasets depended on which studies provided publicly available data that could be analyzed. The inclusion of patients from our center comprised all patients who received immunotherapy during a certain period of time
Data exclusions	no data were excluded from the analyses
Replication	Based on the data and methods (code) provided in this study, the same research results can be obtained.
Randomization	In this study, the dataset was divided into training set and validation set based on its sources. The training set included an Asian patient cohort and a Caucasian patient cohort, while other datasets were used as the validation set.
Blinding	The study was conducted retrospectively, all of the data analyzed was readily available and visible to the researchers during the analysis process

Reporting for specific materials, systems and methods

We require information from authors about some types of materials, experimental systems and methods used in many studies. Here, indicate whether each material, system or method listed is relevant to your study. If you are not sure if a list item applies to your research, read the appropriate section before selecting a response.

Materials & experimental systems

n/a	Involved in the study	
☐	☒	Antibodies
☒	☐	Eukaryotic cell lines
☒	☐	Palaeontology and archaeology
☒	☐	Animals and other organisms
☐	☒	Clinical data
☒	☐	Dual use research of concern
☒	☐	Plants

Methods

n/a	Involved in the study	
☒	☐	ChIP-seq
☐	☒	Flow cytometry
☒	☐	MRI-based neuroimaging

Antibodies

Antibodies used	The specific antibodies used, sourced from eBioscience, included CD3-efluor 450 (OKT3) (catalog: 48-0037-42), CD8-APC (RPA-T8) (catalog: 17-0088-42), and CD279 (PD-1)-PE (MIH4) (catalog: 12-9969-42). CD8-FITC (T8) was purchased from MBL (catalog: K0227-4). 4-1BB-APC (4B4-1) was purchased from biolegend (catalog: 309810).
Validation	The specific antibodies used, sourced from eBioscience, included CD3-efluor 450 (OKT3) (catalog: 48-0037-42), CD8-APC (RPA-T8) (catalog: 17-0088-42), and CD279 (PD-1)-PE (MIH4) (catalog: 12-9969-42). CD8-FITC (T8) was purchased from MBL (catalog: K0227-4). 4-1BB-APC (4B4-1) was purchased from biolegend (catalog: 309810).

Clinical data

Policy information about clinical studies

All manuscripts should comply with the ICMJE guidelines for publication of clinical research and a completed CONSORT checklist must be included with all submissions.

Clinical trial registration	Na
Study protocol	The study protocol is outlined in the manuscript's methodology section.
Data collection	All patients from our center were enrolled and received treatment with anti-PD-(L)1 monotherapy between March 14, 2017 and May 2, 2018.
Outcomes	The efficacy was determined by the Response Evaluation Criteria in Solid Tumors (RECIST) version 1.1, including CR,PR, SD, and progressive disease. Disease control indicated the patients who achieved disease control (i.e., CR, PR, or SD according to RECIST version 1.1). PFS was defined as the time from the start of immunotherapy until either objective disease progression (assessed by an investigator using RECIST version 1.1) or death from any cause. OS was defined as the time from the start of immunotherapy until death from any cause.

Plants

Seed stocks	Na
Novel plant genotypes	Na
Authentication	Na

Flow Cytometry

Plots

Confirm that:

☒ The axis labels state the marker and fluorochrome used (e.g. CD4-FITC).

☒ The axis scales are clearly visible. Include numbers along axes only for bottom left plot of group (a 'group' is an analysis of identical markers).

☒ All plots are contour plots with outliers or pseudocolor plots.

☒ A numerical value for number of cells or percentage (with statistics) is provided.

Methodology

Sample preparation	PBMCs were isolated from 10 ml of fresh peripheral blood containingan anticoagulant by density gradient centrifugation using LymphoPrep(Progen, Heidelberg, Germany).
Instrument	BD FACSAria II
Software	Flowjo
Cell population abundance	The number of sorted PD-1+CD8+T cells was approximately 10^5 in one sample.
Gating strategy	Single mononuclear cells were selected by FSC and SSC. Within the single cells, live cells were gated by a live/dead cell stain. Subsequently, anti-CD8-APC and anti-PD-1-PE positive were selected.

☒ Tick this box to confirm that a figure exemplifying the gating strategy is provided in the Supplementary Information.
